# The Anti-Inflammatory Effects of Fermented Herbal Roots of *Asparagus cochinchinensis* in an Ovalbumin-Induced Asthma Model

**DOI:** 10.3390/jcm7100377

**Published:** 2018-10-22

**Authors:** Jun Young Choi, Ji Eun Kim, Jin Ju Park, Mi Rim Lee, Bo Ram Song, Ji Won Park, Mi Ju Kang, Hee Seob Lee, Hong Joo Son, Jin Tae Hong, Dae Youn Hwang

**Affiliations:** 1 College of Natural Resources and Life Science/Life and Industry Convergence Research Institute, Pusan National University, Miryang 627-706, Korea; junyoung4113@naver.com (J.Y.C.); prettyjiunx@naver.com (J.E.K.); jjpearl0005@naver.com (J.J.P.); rloverim@naver.com (M.R.L.); 94sbr@naver.com (B.R.S.); pjw08260824@naver.com (J.W.P.); beautifulbead@naver.com (M.J.K.); shjoo@pusan.ac.kr (H.J.S.); 2 College of Human Ecology, Pusan National University, Busan 609-735, Korea; heeseoblee@pusan.ac.kr; 3 College of Pharmacy, Chungbuk National University, Chungju 361-763, Korea; jinthong@chungbuk.ac.kr

**Keywords:** asthma, *Asparagus cochinchinensis*, fermentation, airway inflammation, airway remodeling, cholinergic regulation

## Abstract

*Introduction:* Roots of *Asparagus cochinchinensis*, which have pharmacologically active ingredients, have received great attention because they show good therapeutic effects for various inflammatory diseases without specific toxicity. This study investigated the anti-asthmatic effects of a butanol extract of *Asparagus cochinchinensis* roots that had been fermented with *Weissella cibaria* (BAW) and its possible underlying cholinergic regulation. *Methods:* Alterations of the anti-asthmatic markers and the molecular response factors were measured in an ovalbumin (OVA)-induced asthma model after treatment with BAW. *Results:* Treatment with BAW decreased the intracellular reactive oxygen species (ROS) production in lipopolysaccharides (LPS) activated RAW264.7 cells. The results of the animal experiments revealed lower infiltration of inflammatory cells and bronchial thickness, and a significant reduction in the number of macrophages and eosinophils, concentration of OVA-specific IgE, and expression of Th2 cytokines in the OVA + BAW treated group. In addition, a significant recovery of goblet cell hyperplasia, MMP-9 expression, and the VEGF signaling pathway was observed upon airway remodeling in the OVA + BAW treated group. Furthermore, these responses of BAW were linked to recovery of acetylcholine esterase (AChE) activity and muscarinic acetylcholine receptor (mAChR) M3 downstream signaling pathway in epithelial cells, smooth muscle cells, and afferent sensory nerves of OVA + BAW-treated mice. *Conclusion:* Overall, these findings are the first to provide evidence that the therapeutic effects of BAW can prevent airway inflammation and remodeling through the recovery of cholinergic regulation in structural cells and inflammatory cells of the chronic asthma model.

## 1. Introduction

Asthma is a chronic and complex inflammatory disease of the lung airways that is characterized by hyper-responsiveness, mucus hypersecretion, reversible obstruction, overexpression of Th2-mediated cytokines, and remodeling in the lung [1,2,3,4]. Immunodeficiency, delayed puberty, adrenal insufficiency, and growth failure are some of the adverse side effects associated with the commonly administered anti-asthmatic drugs, including glucocorticoids, antihistamines, and immunosuppressants [5,6,7,8]. Therefore, recent studies have focused on identifying natural products with pharmacologically active ingredients to provide new remedies without unwanted side effects [9]. Several extracts of natural herbs, including Samsoeum [10], *Erythronium japonicum* in East Asia [11], *Trigonella foenum-graecum* in Central Asia and Eastern Europe [12], *Echinodorus scaber* Rataj in the western hemisphere [13], and *Urtica dioica* in Asia, Europe, Africa, and Americas [14], attenuate airway inflammation of allergic asthma in an ovalbumin (OVA)-induced model. However, fermented natural herbs have exclusively been investigated for their therapeutic effect in an asthma model, despite enhancing the levels of functional components, breakdown of substances, and chemical transformations of certain constituents [15].

Although there is scientific evidence of the anti-inflammatory effects of *A. cochinchinensis* root extracts, most studies have investigated unfermented products. An aqueous extract significantly inhibited the expression of tumor necrosis factor (TNF)-α and pro-inflammatory cytokines in mouse astrocytes stimulated with lipopolysaccharide (LPS)- and substance P [16]. Another study confirmed that the ethanolic extract of *A. cochinchinensis* roots suppress the nitric oxide (NO) production in LPS-treated BV-2 microglial cells and decrease the progression of cutaneous inflammation in animal models treated with 12-O-tetradecanoyl-phorbol-13-acetate (TPA) [17]. The saponin-enriched extracts of unfermented *A. cochinchinensis* (SEAC) decreased skin inflammation of IL-4/Luc/CNS-1 transgenic (Tg) mice treated with phthalic anhydride (PA) [18]. Moreover, administering a combination of *A. cochinchinensis* roots and six medicinal herbs effectively reduced the number of immune cells in the bronchoalveolar lavage fluid of mice treated with a cockroach allergen [19]. Unfermented products of *A. cochinchinensis* also suppressed the number of immune cells in the bronchoalveolar lavage fluid (BALF), the concentration of OVA-specific IgE, the infiltration of inflammatory cells, the bronchial thickness, and the level of inflammatory mediators in an OVA-induced asthma model, while they contained 57.2 mg/g of crude saponin, 88.5 µg/g of total phenols, and 102.1 µg/g of total flavonoids [20]. After fermentation, the root extracts of *A. cochinchinensis* (BAW) contained an enhanced concentration of a steroidal saponin compounds (126.6%), total phenols (9.3%), and protodioscine compared with their unfermented products [21]. Evaluating the anti-inflammatory activities of fermented products of *A. cochinchinensis* root in LPS-stimulated macrophage cells (RAW264.7 cells) revealed that the inflammatory responses were significantly suppressed in response to BAW treatment via regulation of the expression of inflammatory cytokines and the iNOS-mediated COX-2 induction pathway [21]. These results provide the possibility that airway inflammation and remodeling in lung tissues are effectively inhibited by BAW in the OVA-induced asthma model, although no studies have shown a direct role of these products in anti-asthmatic effects or revealed their mechanism of action.

Therefore, we investigated the ability of BAW to prevent airway inflammation as well as its mechanism of action and remodeling activity using an OVA-induced mouse asthma model. The present study provides the first evidence of a correlation between anti-asthmatic effects of BAW and cholinergic regulation in the airways of the OVA-induced asthma model. Our results show that BAW may be a potential drug for treatment of patients with severe airway changes.

## 2. Materials and Methods

### 2.1. Preparation of BAW

BAW was prepared as previously described [21]. Briefly, the fresh roots of *A. cochinchinensis* were obtained from Gochang National Agriculture Cooperation in Gochang-gun, Korea and dried at 60 °C for 5–6 h. The dried roots of *A. cochinchinensis* roots (WPC-14-003) were then placed as voucher specimens in the functional materials bank of the PNU-Wellbeing RIS Center. Additionally, these roots were identified and confirmed by Dr. Shin Woo Cha at the National Institute of Horticultural & Herbal Science, Eumseong-gun, Korea.

The aqueous fractions of unfermented *A. cochinchinensis* (UnF) were prepared by powdering 20 g of the dried roots of *A. cochinchinensis*, after which they were subjected to 2.5 h of hot water extraction in 1.2 L of dH_2_O. On completion of the aqueous extraction, these solutions were filtered through Whatman No.2 filter paper (Whatman, Brentford, UK), after which they were subjected to evaporation using a vacuum evaporator (EYELA, N-1100 series, Tokyo, Japan). This process of hot water extraction yielded 60.7% UnF, which was freeze-dried and used for fermentation at a later stage.

To prepare the fermented samples, UnF powder was completely dissolved in dH_2_O (pH 5.3) to 1% (*w*/*v*), after which the mixture solution was sterilized at 121 °C for 15 min using an autoclave (Hashin medical Co., Seoul, Korea). *W. cibaria* pre-cultivated in lactobacilli MRS broth (Difco Laboratories, Detroit, MI, USA) with a cell density of 10^7^ CFU/mL (OD600 = 0.1) were inoculated (5% (*v*/*v*)) into the UnF mixture. Following incubation at 37 °C and 200 rpm for 4.3 days, the fermented *A. cochinchinensis* products of *W. cibaria* (FPW) was harvested by centrifugation at 12,000× *g* for 10 min.

**Figure 1:**
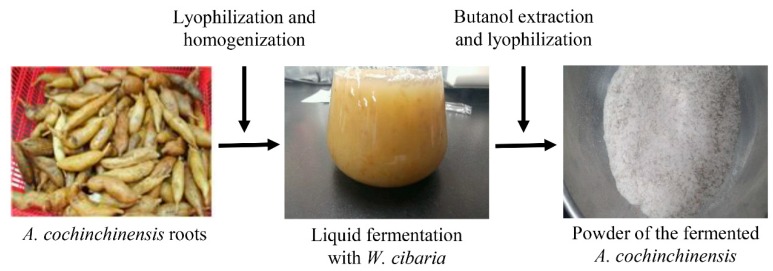
Preparation of BAW sample. BAW was obtained from the root of *A. cochinchinensis* after fermentation with *W. cibaria*, extraction with butanol, and lyophilization as described in the materials and methods.

To collect n-butanol fractions of FPW (BAW), FPW were suspended in an equal volume of butanol, after which the butanol phase was collected from each mixture by centrifuging at 12,000× *g* for 10 min. The resulting phases were then concentrated under a rotary vacuum evaporator to obtain the final extracts (Figure 1). The aqueous extract of unfermented *Platycodon grandiflorus* (PG), a positive control, was successively extracted with hot water at 120 °C for 45 min at a fixed liquor ratio (solid powder of PG: dH_2_O ratio at 75 g:500 mL). Dexamethasone (Dex) was procured from Sigma-Aldrich Co. (St. Louis, MO, USA).

### 2.2. Measurement of Bioactive Compounds in BAW

The concentration of the two bioactive compounds, total phenols, and crude saponins in BAW were measured as described in previous studies [20]. First, the amount of total phenols was analyzed by the Folin–Ciocalteu method. Briefly, BAW (20 μL) and 0.2 N Folin–Ciocalteu reagent (100 μL) were mixed with 20% sodium carbonate (300 μL), then incubated at room temperature for 2 h. The absorbance of this mixture was subsequently measured at 765 nm in an enzyme-linked immunosorbent assay (ELISA) Plate Reader (Molecular Devices, San Jose, CA, USA). A standard calibration curve generated with gallic acid was used to determine the total phenolic content, which was reported as mg gallic acid per gram of BAW.

The amount of crude saponins was measured according to our previous studies [21]. Briefly, BAW were suspended in 30 mL water, then extracted with n-butanol (30 mL) three times. The resulting layer was subsequently concentrated and lyophilized using circulation extraction equipment (IKA Labortechnik, Seoul, Korea).

### 2.3. High Performance Liquid Chromatography (HPLC) Analysis

The saponin protodioscin in BAW was analyzed using an ILC 3000 HPLC system (Interface Engineering Co. Ltd., Seoul, Korea) equipped with a Corona CAD detector (ESA Biosciences, Inc., Chelmsford, MA, USA). Chromatographic separation for the quantification was performed on a CapCell PAK C18 ACR column (4.6 × 250 mm, particle size 5 μm; Shiseido Co., Ltd., Tokyo, Japan) using a mobile phase that consisted of solvent A (dH_2_O) and solvent B (acetonitrile). Sample analysis was conducted at an applied flow rate of 1.0 mL/min using compressed nitrogen as the nebulizer gas. During analysis, the gas flow rate and gas pressure were maintained at 1.53 L/min and 35 ± 2 psi, respectively. The output signal of the detector was recorded with the Clarity™ chromatography software (DataApex, Prague, Czech Republic).

### 2.4. Scavenging Activity of Free Radical

The scavenging capability of the 2,2-diphenyl-1-picrylhydrazyl (DPPH) radical was measured at eight different concentrations (7.8 to 600 μg/mL) of BAW according to the method described in a previous study [22]. Briefly, each sample of BAW (100 µL) was mixed with 100 µL of 0.1 mM DPPH (Sigma-Aldrich; Merck KGaA, Darmstadt, Germany) prepared in 95% EtOH solution. After 30 min of incubation at room temperature, the absorbance of the reaction mixture was recorded using a Versa-max plate reader (Molecular Devices, Sunnyvale, CA, USA) at a wavelength of 517 nm. The percent drop in the absorbance, relative to that in the control was used to determine the DPPH radical scavenging activity of the BAW. The concentration of BAW resulting in a 50% loss in DPPH activity was determined to be the IC_50_.

### 2.5. Analysis of Intracellular Reactive Oxigen Species (ROS) Level

The intracellular ROS levels in RAW264.7 cells were evaluated using a 2′,7′-dichlorofluorescein diacetate (DCFH-DA; Sigma-Aldrich; Merck KGaA, Darmstadt, Germany) fluorescent probe. The cell permeable DCFH-DA was deacetylated using intracellular esterases to form non-fluorescent 2′,7′-dichlorodihydrofluorescein (DCFH). In the presence of ROS, the DCFH was intracellularly converted to the highly fluorescent 2′,7′-dichlorofluorescein (DCF). Briefly, six-well plates were seeded with RAW364.7 cells at a density of 5 × 10^5^ cells/2 mL, then cultured in the presence of two concentrations of BAW for 2 h in a 37 °C incubator. After washing with 1× PBS, the cells were treated with either vehicle (DMSO) or BAW (100 µg/mL) for 2 h, exposed to LPS (1 μg/mL) for another 24 h, then incubated with 25 μM DCFH-DA for 30 min at 37 °C. Finally, the cells were observed at 200× magnification for green fluorescence using a fluorescence microscope (Eclipse TX100, Nikon, Tokyo, Japan).

### 2.6. Design of Animal Experiment

The Institutional Animal Care and Use Committee at Pusan National University (PNU-IACUC) reviewed and approved the ethical and scientific care procedures for the animal experiments (Approval Number PNU-2015-0779). BALB/c mice (6-week-old females) were provided by Samtako BioKorea Co. (Osan, Korea). Prior to the experiment, they were acclimatized to the experimental environment for at least 1 week. During the experiment, mice were given *ad libitum* access to a standard irradiated chow diet (Samtako-Bio Korea Co., Osan, Korea) and autoclaved water. All BALB/c mice were maintained in a specific pathogen-free state (SPF) under a strict light cycle (lights on at 08:00 h and off at 20:00 h) at 23 ± 2 °C and 50 ± 10% relative humidity throughout the animal study. The Laboratory Animal Resources Center at Pusan National University, which is accredited by the AAALAC International according to the National Institutes of Health guidelines (Accredited Unit Number; 001525), was used for breeding of all mice.

Asthma was induced in BALB/c mice via OVA administration as previously described [23,24]. Briefly, BALB/c mice (6-week-old female, *n* = 48) were randomly divided into either a No-treated group (untreated controls, *n* = 8) or an ovalbumin (OVA)-treated group (*n* = 40). The experimental procedures for OVA-induced asthma consisted of sensitization for 20 days and challenge for 3 days. At day 1 and day 14, sensitization was achieved by intraperitoneal injection with OVA (20 μg) (albumin from chicken, Sigma-Aldrich; Merck KGaA, Darmstadt, Germany) emulsified with aluminum hydroxide (Alum, Sigma-Aldrich; Merck KGaA, Darmstadt, Germany) in 200 µL 1× PBS solution. From days 21 to 23, all sensitized mice were subject to inhalation with 2% OVA aerosol for 30 min through a nebulizer (NE-C28, Omron, Tokyo, Japan). The OVA-treated group was further assigned into one of following groups: vehicle-treated group (OVA + Vehicle, *n* = 8), Dex-treated group (OVA + Dex, *n* = 8), PG-treated group (OVA + PG, *n* = 8), low concentration of BAW-treated group (OVA + BAWLo, *n* = 8), and high concentration of BAW-treated group (OVA + BAWHi, *n* = 8). These groups were administered orally for 6 days from 3 days prior to the first challenge. The OVA + BAW treated groups were orally administered 250 and 500 mg/kg body weight of BAW (BAWLo and BAWHi, respectively) for 6 days. Moreover, the OVA + Vehicle group, OVA + Dex group, and OVA + PG group were orally administered the same volume of vehicle solution (0.5% Tween-20), Dex solution (3 mg/kg body weight), and PG (250 mg/kg body weight) for 6 days. All animals were euthanized using CO_2_ gas at 48 h after the final treatment, at which time tissue samples to be used in the analyses were collected and stored at −70 °C.

### 2.7. Enumeration of Total Cells in Bronchoalveolar Lavage Fluid (BALF)

After anesthetizing the BALB/c mice with alfaxan (Alfaxalone^®^, Jurox Pty Ltd., Hunter Valley, Australia), the mouse lungs were lavaged three times with cold 1× PBS (yield: 80%, total volume = 0.8 mL). After centrifugation of BALF at 2,000× *g* for 5 min at 4 °C, the supernatant was collected for ELISA analysis and the pellets were used for cell analysis. Total cells of the BALF pellet were attached to a slide glass using a cytospin (5 min, 500 rpm, Hanil Electric, Wonju, Korea), then fixed in methanol for 30 s. These slides were subsequently processed in May–Grunwald solution (Sigma-Aldrich; Merck KGaA, Darmstadt, Germany) for 5 min, then in Giemsa solution (Sigma-Aldrich; Merck KGaA, Darmstadt, Germany) for 12 min. After rinsing three times, the slides were covered, after which immune cells were counted under light microscopy at 400× magnification.

### 2.8. Enzyme-Linked Immunosorbent Assay (ELISA) for IL-4 in BALF and Serum

The concentration of mouse IL-4 was measured in both BALF and serum using an IL-4 ELISA kit (BioLegend, San Diego, CA, USA) following the manufacturer’s recommendations. Mixtures of BALF (or serum, 50 µL each) and assay buffer (50 µL) were reacted with anti-IL-4 antibody in a 96-well plate for 2 h at room temperature. After removal of unbound proteins, detection antibody solution (100 µL), and avidin–horseradish peroxidase (HRP) D solution (100 µL) was sequentially added to the wells, after which samples were incubated at room temperature for 30 min. Finally, these mixtures were reacted with substrate solution (100 µL) for 15 min, after which the reaction was stopped with blocking solution (100 µL). The absorbance of the mixture was then read at 450 nm using a Versa-max plate reader (Molecular Devices, San Jose, CA, USA).

### 2.9. Detection of OVA-Specific IgE Concentration

Assessment of the OVA-specific IgE concentration in the BALF and serum of mice was conducted using an ELISA kit (BioLegend Inc., San Diego, CA, USA). Briefly, a mixture of BALF (or serum, 50 µL per each) and assay buffer (50 µL) was reacted with OVA-specific IgE antibody in each well for 2 h while shaking at room temperature. Following removal of unbound proteins, detection antibody solution (100 µL) and Avidin-HRP D solution (100 µL) was sequentially added to each well, then incubated for 1 h with shaking at room temperature. An enzyme reaction was initiated by adding the substrate solution and terminated by adding 2 M H_2_SO_4_ solution. The absorbance of the mixture was then read at 450 nm with a Versa-max plate reader (Molecular Devices, San Jose, CA, USA).

### 2.10. Histopathological Analysis

To exactly measure the epithermal thickness of the bronchial tree, the histopathological features were analyzed in the same region of the lung. Briefly, the right lungs were collected from mice of a subset group, then fixed in a 10% neutral buffered formalin for 48 h. Next, the middle lobes were exactly trimmed from the fixed tissues and embedded in the same direction and position to make paraffin blocks. After sectioning the block into 4 μm thick slices, the lung section forms #50 to #70 were collected from the whole series of the section. The sections were then stained with hematoxylin and eosin (H&E) (Sigma-Aldrich; Merck KGaA, Darmstadt, Germany), after which the same region on the positioning of the bronchus within the bronchial tree were microscopically examined at 400× magnification for histopathological features. The smooth muscle thickness and epithelial thickness in the bronchial tubes were determined using the Leica Application Suite (Leica Microsystems, Wetzlar, Germany). In addition, the degree of cell infiltration in the airway was scored in a double-blind screen by two independent investigators based on a previous study [25]. The peri-bronchiole and peri-vascular inflammation were evaluated using a scoring of 0–5 (0, no cells; 1, a few cells; 2, a ring of cells 1 cell-layer deep; 3, a ring of cells 2–4 cells deep; 4, a ring of cells; and 5, cells deep). For each mouse, five airway sections that were randomly distributed through the left lung were analyzed, and their average scores were calculated.

Periodic acid–Schiff (PAS) staining detected the goblet cell hyperplasia for mucus production. Deparaffinization and dehydration of the lung sections were followed by oxidization of the samples in periodic acid solution for 5 min, washing, and depositing in Schiff reagent for 15 min. After washing these sections, the lung tissue was stained with hematoxylin solution for 30 s (Sigma-Aldrich; Merck KGaA, Darmstadt, Germany), then observed under light microscopy at 400× magnification for goblet cell hyperplasia and sub-epithelial fibrosis. The mucus score was also measured by four independent investigators in a double-blind study analysis based on four different random locations using a microscope as follows: 0, no goblet cells; 1, <20% of the epithelium; 2, 20–40% of the epithelium; 3, 40–60% of the epithelium; 4, 60–80% of the epithelium; and 5, >80% of the epithelium [25].

### 2.11. Quantitative Real-Time Polymerase Chain Reaction (PCR) Analysis for Cytokine Gene Expression

Quantitative real-time PCR assessed the relative quantities of mRNA for IL-4, IL-13, TNF-α, and IL-1β. Total RNA molecules were isolated from frozen lung tissues using RNA Bee solution (Tet-Test Inc., Friendswood, TX, USA). After quantification of the RNA concentration, the complement DNA (cDNA) was synthesized using a mixture of oligo-dT primer (Invitrogen, Carlsbad, CA, USA), dNTP and reverse transcriptase (Superscript II, 18064-014, Invitrogen; Thermo Fisher Scientific, Inc., Waltham, MA, USA). Q-PCR was then conducted using a cDNA template and 2× Power SYBR Green (TOYOBO Co., Osaka, Japan) as described in previous studies [26]. The reaction cycle at which PCR products exceeded this fluorescence intensity threshold during the exponential phase of PCR amplification was considered as the threshold cycle (CT).

### 2.12. AChE Activity Analysis

The AChE activity was determined using an Acetylcholinesterase Assay Kit (Abcam, Cambridge, UK) according to the manufacturer’s protocols. Briefly, the lung tissue of each mouse was homogenized in PRO-PREP protein extraction solution (1.0 mM PMSF, 1.0 mM EDTA, 1.0 µM pepstatin, 1.0 µM leupeptin, and 1.0 µM aprotinin)(iNtRON Biotechnology Inc., Seoul, Korea), after which the homogenates were stored at −70 °C until analysis. The sample or standards and ACh reaction mixture were then incubated on a 96-well plate for 10 min at room temperature while protected from the light. Color alterations were read using a Vmax plate reader (Molecular Devices, Sunnyvale, CA, USA) at 410 nm.

### 2.13. Western Blot Analysis

After collecting total tissue homogenates using the PRO-PREP^TM^ Solution (iNtRON Biotechnology Inc., Sungnam, Korea), the proteins were separated on 10% sodium dodecyl sulfate-polyacrylamide gel electrophoresis (SDS-PAGE) for 2 h at 100 V, then transferred to a nitrocellulose membrane (GE Healthcare, Little Chalfont, UK) at 40 V for 2 h. The proteins on membranes were subsequently hybridized with specific primary antibodies overnight at 4 °C: anti-MMP-9 (1:1000, Santa Cruz Biotechnology, Santa Cruz, CA, USA), anti-VEGF (1:1000, PeproTech, Rocky Hill, NJ, USA), anti-ERK (1:1000, Cell Signaling Technology, Danvers, MA, USA), anti-p-ERK (1:1000, Cell Signaling Technology), anti-MLC (1:1000, Abcam, Cambridge, UK), anti-p-MLC (1:1000, Abcam), anti-mAChR M3 (1:1000, Abcam), anti-G protein α (1:1000, Abcam), anti-PKC (1:1000, Cell Signaling Technology), anti-p-PKC (1:1000, Cell Signaling Technology), anti-PI3K (1:1000, Cell Signaling Technology), anti-p-PI3K (1:1000, Cell Signaling Technology), and anti-β-actin (1:1000, Sigma-Aldrich; Merck KGaA, Darmstadt, Germany). After removing the unbound antibodies, the membranes were incubated with horseradish peroxidase-conjugated anti-secondary antibody for 1 h at room temperature. Finally, each specific band was detected using an enhanced chemiluminescence reagent plus kit (Amersham Biosciences, Corston, UK). The Image Analyzer System (Fluorchem FC2, Alpha Innotech, CA, USA) was used to quantify the results, which are expressed as the fold-increase over control values.

### 2.14. Statistical Analysis

Statistical significance was evaluated using one-way analysis of variance (ANOVA) (SPSS for Windows, Release 10.10, Standard Version, Chicago, IL, USA) followed by Tukey’s post hoc *t*-test for multiple comparisons. All values were expressed as the means ± SD and a *p* < 0.05 was considered statistically significant.

## 3. Results

### 3.1. Bioactive Components and Antioxidant Activity of BAW

**Figure 2:**
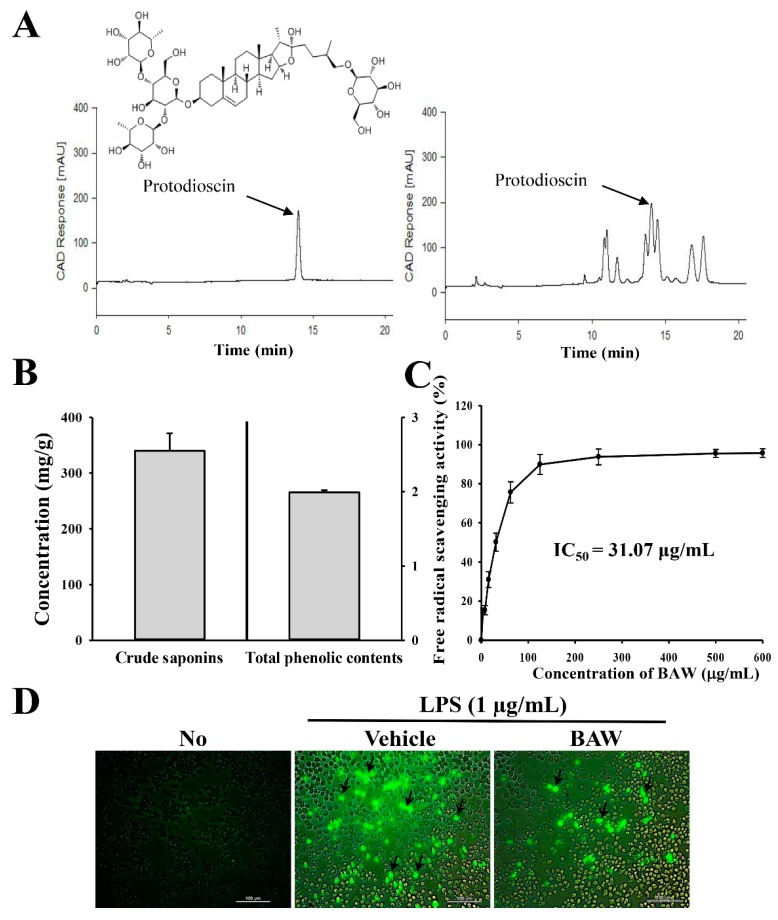
Antioxidant properties of BAW. (**A**) Chromatograms of protodioscin were obtained by performing high-performance liquid chromatography (HPLC) of BAW. The peak height/area reflects the concentration of protodioscin in BAW. (**B**) Crude saponins and total phenolic contents were analyzed in mixtures containing different concentrations of BAW. The contents of crude saponins were calculated using the following equation: crude saponins (mg/g) = A − B/S, where, A is the dry weight of the n-butanol layer (mg), B is the weight of the flask (mg), and S is the weight of the BAW extract (g). (**C**) Free radical scavenging activity of BAW. DPPH radical scavenging activity was assayed in a mixture containing 0.1 mM DPPH and varying concentrations of BAW (0−600 μg/mL). The data represents the means ± standard deviation (SD) (*n* = 8). DPPH, 2,2-diphenyl-1-picrylhydrazyl radical; IC_50_, half maximum inhibitory concentration. (**D**) Green fluorescence of ROS in cells co-treated with LPS and BAW was observed using fluorescence microscopy at 200× magnification. Arrows indicate cells stained with DCFH-DA.

As shown in Figure 2B, BAW contained high concentrations of two bioactive components related to anti-inflammatory activity, crude saponins and total phenols (340 ± 31.4 mg/g and 1.99 ± 0.03 mg/g, respectively). Among the crude saponins, protodioscin generated a sharp peak in the chromatogram of HPLC and was determined to be present at a level of 34.5 µg/mg in BAW (Figure 2A). Furthermore, the increase in scavenging activity of BAW against DPPH radicals was rapid during the early stages, but was slower in the latter stage, with an IC_50_ of 31.07 μg/mL (Figure 2C). Intracellular ROS analysis revealed a significant increase in the number of cells stained with DCFH in the LPS + Vehicle-treated group relative to the No-treated group. However, these numbers dramatically decreased in cells pretreated with LPS + BAW (Figure 2D). These results provide strong evidence that BAW encompassed high anti-oxidative activity, which was obtained because of the high levels of anti-oxidant compounds as well as inhibition of increases in intracellular ROS production.

### 3.2. BAW Suppress the Influx of Leukocytes in BALF of OVA-Induced Asthma Model

**Figure 3:**
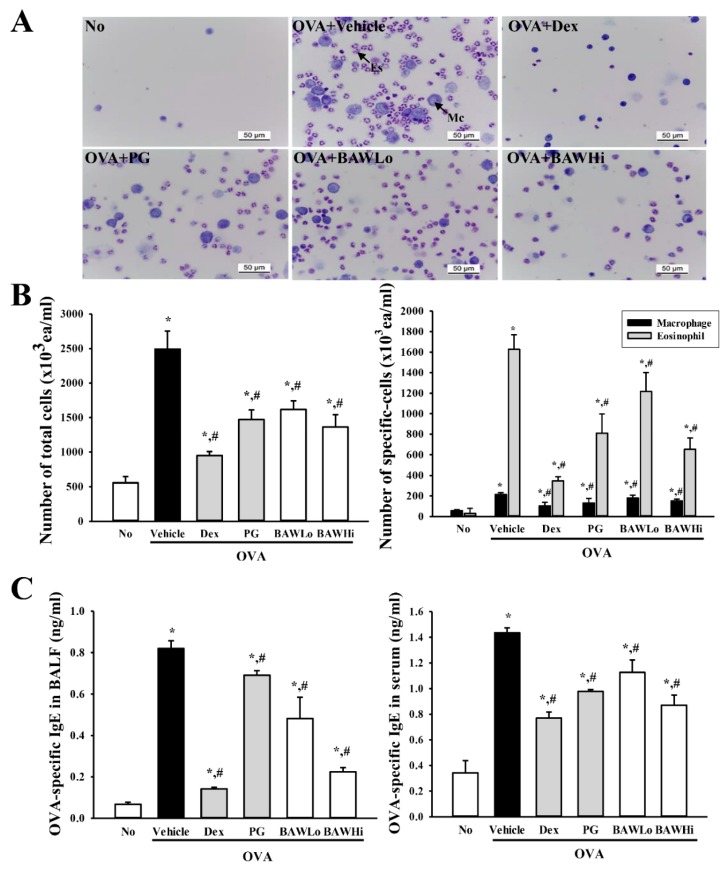
Suppression effects of BAW on the influx of inflammatory cells and IgE concentration in BALF. (**A**) Total cells were collected from the BALF of lungs using centrifugation and measured repeatedly. These cells were then suspended in PBS and applied to a slide by cytospinning, stained with May–Giemsa solution, and observed under a light microscope at 400× magnification. (**B**) The number of total cells, eosinophils, and macrophages was determined by counting within a 1 mm^2^ area. (**C**) The concentration of OVA-specific IgE was quantified in BALF and serum using an enzyme-linked immunosorbent assay with a minimum detectable concentration of 20.7 pg/mL. Es, Eosinophil; Mc, Macrophage. The data shown represents the means ± SD (*n* = 8). * indicates *p* < 0.05 compared to the No-treated group. # indicates *p* < 0.05 compared to the OVA + Vehicle treated group.

To investigate the suppression effects of BAW against the influx of leukocytes in the bronchoalveolar lavage, we determined the number of total cells, macrophages, and eosinophils in BALF of OVA + BAW treated mice. A significant enhancement of all cells was observed in the OVA + Vehicle group compared to the No-treated group, reflecting OVA-challenged airway inflammation. However, the treatment groups OVA + Dex, OVA + PG, and OVA + BAW showed a significant decline in the number of total cells, macrophages, and eosinophils in BALF when compared with those of the OVA + Vehicle group. The suppressive effects of BAW were very similar to those of Dex, which was used as a positive control (Figure 3A,B). These findings indicate that BAW treatment suppressed the influx of leukocytes into the bronchoalveolar fluid after OVA inhalation.

### 3.3. BAW Effect on IgE Concentration in Serum and BALF of OVA-Induced Asthma Model

Altered IgE levels are considered key evidence for the improvement of pathological symptoms in OVA-treated BALB/c mice [1,4]. Therefore, to evaluate the suppression of OVA-specific IgE secretion from B cells, we measured the IgE levels in the BALF and serum of OVA-sensitized BALB/c mice treated with vehicle, Dex, PG, and BAW. We observed higher levels of the OVA-specific IgE in the OVA + Vehicle treated group, suggesting successful OVA induction in the asthma model. Conversely, the OVA-specific IgE levels decreased in the OVA + Dex, OVA + PG, and OVA + BAW treated groups when compared with the OVA + Vehicle treated group (Figure 3C). Furthermore, this suppressive effect was greater in BALF than in serum. Therefore, the present results indicate that BAW treatment successfully inhibits the production of OVA-specific IgE through the induction of B-cell isotype switching in the OVA-induced asthma model.

### 3.4. Alterations in Inflammatory Cell Infiltration and Epithelial Damage of OVA-Induced Asthma Model Treated with BAW

**Figure 4:**
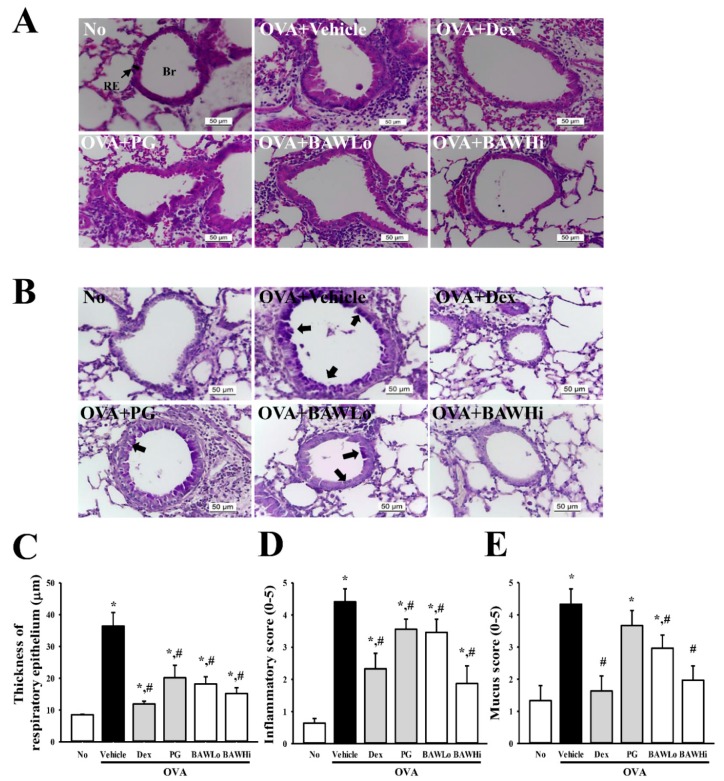
Histopathology in lung tissue of OVA-induced asthma model. (**A**) Histological alteration of lung tissue. The epithelial thickness and infiltration of inflammatory cells in the peribronchiolar region were observed in lung tissue stained with H&E solution at 400× magnification. (**B**) Goblet cell hyperplasia. Mucus production was observed in Periodic acid–Schiff (PAS) stained lung tissue at 400× magnification. (**C**) Quantification of epithelial thickness. Alterations in epithelial thickness of lung tissue were measured using the Leica Application Suite. (**D**) Inflammatory infiltration score of airway. The degree of cell infiltration in the airway was scored in a double-blind screen by two independent investigators based on a previous study [25]. The peri-bronchiole and peri-vascular inflammation were evaluated using a score of 0–5: 0, no cells; 1, a few cells; 2, a ring of cells 1 cell-layer deep; 3, a ring of cells 2–4 cells deep; 4, a ring of cells; and 5, cells deep. For each mouse, five airway sections that were randomly distributed through the left lung were analyzed, and their average scores were calculated. (**E**) Mucus score of airway. The mucus score was measured by four independent investigators in a double-blind study based on four different random locations using a microscope: 0, no goblet cells; 1, <20% of the epithelium; 2, 20–40% of the epithelium; 3, 40–60% of the epithelium; 4, 60–80% of the epithelium; and 5, >80% of the epithelium [25]. Br, Bronchus; RE, Respiratory epithelium. The data represents the means ± SD (*n* = 8). * indicates a *p* < 0.05 compared to the No-treated group. # indicates a *p* < 0.05 compared to the OVA + Vehicle-treated group. Arrows represent the areas of mucin secretion.

To investigate whether BAW induces recovery from inflammatory cell infiltration and epithelial damage to airways, we studied the changes in the histopathological features of lungs in an OVA-induced asthma model treated with BAW. We observed increased thickness of the respiratory epithelium in lung tissue sections in mice that had been sensitized with OVA (OVA + Vehicle treated group). However, the thickness significantly decreased in the OVA + BAWLo- and OVA + BAWHi-treated groups when compared with the OVA + Vehicle-treated group. Furthermore, there was decreased infiltration of the inflammatory cells in the peribronchiolar region in both BAW-treated groups (Figure 4A,C,D). These results indicate that BAW treatment has the potential to alleviate the epithelial damage and infiltration of the inflammatory cells in airways of the OVA-induced asthma model.

### 3.5. Alteration in the Expression of Key Cytokines in OVA-Induced Asthma Model Treated with BAW

**Figure 5:**
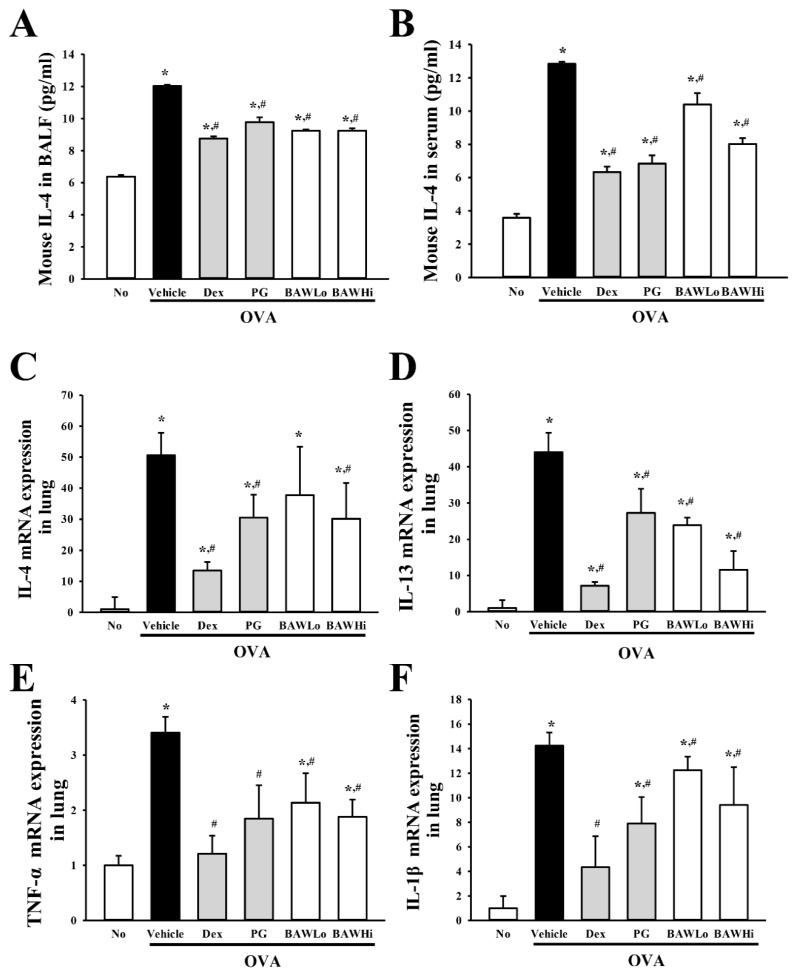
Level of inflammatory cytokines. After collecting the supernatant from BALF and serum from the OVA-induced asthma model were treated with BAW, the concentrations of IL-4 were measured in BALF (**A**) and serum (**B**) using an IL-4 ELISA kit that could detect IL-4 at 0.5 pg/mL. The levels of IL-4 (**C**), IL-13 (**D**), TNF-α (**E**), and IL-1β (**F**) mRNAs in lung tissue were detected using real-time PCR analysis using specific primers. The relative level of mRNA for each specific cytokine was calculated based on the intensity of β-actin mRNA as an endogenous control. The data shown represent the means ± SD (*n* = 8). * indicates *p* < 0.05 compared to the No-treated group. # indicates *p* < 0.05 compared to the OVA + Vehicle-treated group.

To examine the inhibitory effects of BAW on the expression of Th2-like and pro-inflammatory cytokines, the levels of IL-4, IL-13, TNF-α, and IL-1β were measured in the lung tissue, BALF, and serum of an OVA-induced asthma model treated with BAW using real-time PCR and ELISA. For Th2-like cytokines, the mRNA levels of IL-4 and IL-13 were higher in the lung tissue of the OVA + Vehicle-treated group than in the No-treated group. These levels decreased remarkably in the OVA + Dex-, OVA + PG-, OVA + BAWLo-, and OVA + BAWHi-treated groups relative to the OVA + Vehicle-treated group, even though the decrease ratio varied (Figure 5C,D). The decrease pattern for IL-4 mRNA was consistent with the concentration of IL-4 protein in BALF and serum (Figure 5A,B). Similar results were observed for pro-inflammatory cytokines (TNF-α and IL-1β). After BAW treatment, the levels of the two cytokines in the lung tissue decreased significantly in a dose-dependent manner (Figure 5E,F). These results indicate that the BAW treatment inhibited the expression of Th2-like and pro-inflammatory cytokines during airway inflammation in the OVA-induced model.

### 3.6. Alteration in Airway Remodeling of OVA-Induced Asthma Model Treated with BAW

To determine if suppression of airway remodeling is accompanied by therapeutic effects of BAW, we assessed the alterations in the goblet cell hyperplasia, peribronchiolar collagen layer and the expression of angiogenesis regulator in the OVA-sensitized asthma model after treatment with BAW. Following sensitization with OVA, the OVA + Vehicle-treated group had an enhanced mucus score in the bronchial airways of mice when compared to the No-treated group, indicating goblet cell hyperplasia. However, these levels were significantly lower in the OVA + BAWLo- and OVA + BAWHi-treated groups than in the OVA + Vehicle-treated group. Recovery of the goblet cell hyperplasia of the airway was also observed after BAW treatment, although the decrease rate varied in each group (Figure 4B,E).

**Figure 6:**
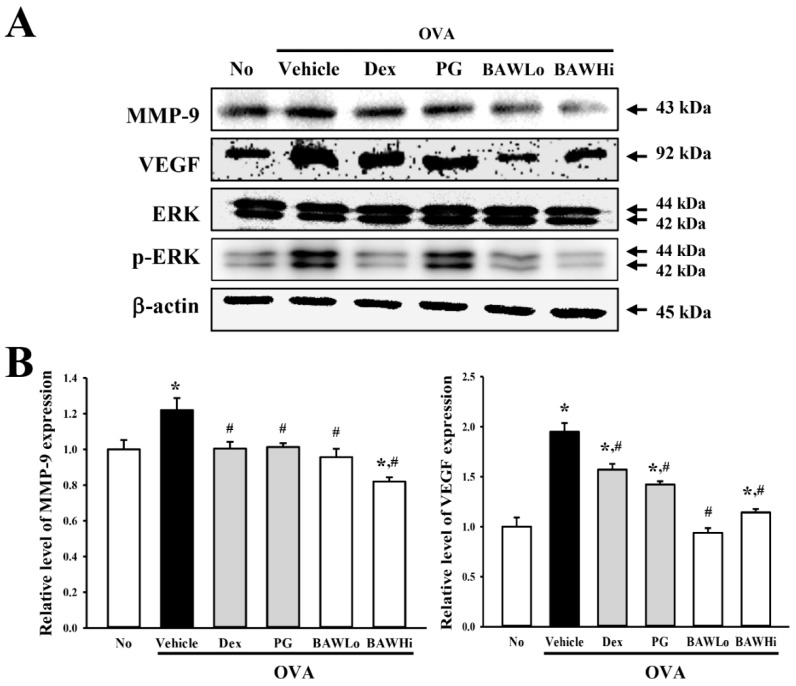
Level of airway remodeling-related factors. (**A**) The expression level of MMP-9 in lung tissue of the OVA + BAW-treated model was measured using specific antibodies. Moreover, changes in the expression of VEGF and the regulator protein under the signaling pathway downstream of VEGF were also examined in lung tissue of a subset group using specific antibodies. (**B**) Band intensities were determined using an imaging densitometer and the expression levels of four proteins were evaluated relative to the intensity of actin bands. The data shown represent the means ± SD (*n* = 8). * indicates *p* < 0.05 compared to the No-treated group. # indicates *p* < 0.05 compared to the OVA + Vehicle-treated group.

We observed similar results when assessing the expression of MMP-9. Elevated expression of MMP-9 is associated with severe asthma and a decline in pulmonary functions [27]. The enhanced MMP-9 expression was recovered in the OVA + BAW-treated groups when compared with the OVA + Vehicle-treated group (Figure 6). Furthermore, we analyzed the expression levels of regulators under the VEGF signaling pathway to investigate the suppression effect of BAW on lung angiogenesis. The expression level of VEGF, which is considered as a stimulator of angiogenesis that causes structural changes in the lung tissue of asthma patients, significantly decreased in the OVA + BAW-treated groups when compared with the OVA + Vehicle-treated group. However, alterations in the VEGF pattern were reflected in entirety only in the ERK phosphorylation among members of the downstream signaling pathway (Figure 6). Therefore, the above results indicate that BAW potentially suppresses airway remodeling by regulating goblet cell hyperplasia, collagenase level, and angiogenesis in airways of the OVA-induced asthma model.

### 3.7. The Mechanism of BAW Action on Cholinergic Regulation of Airway Inflammation and Remodeling

**Figure 7:**
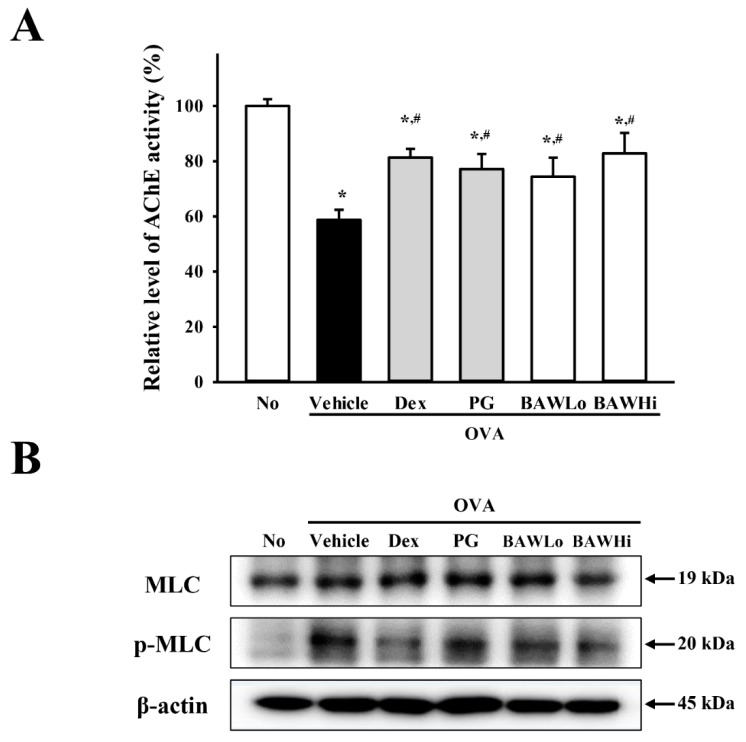

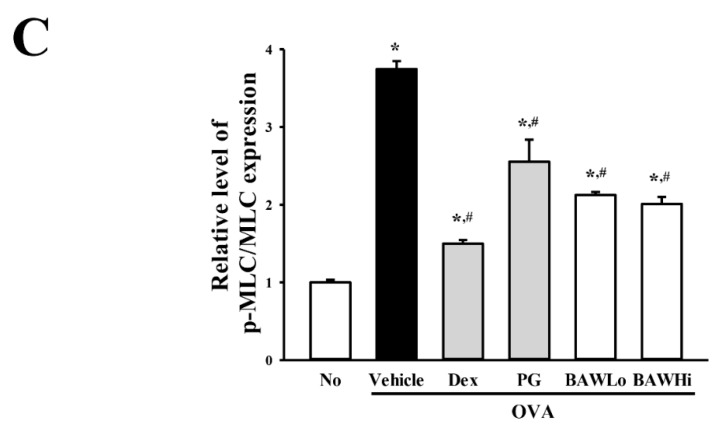
AChE activity and AChR M3 downstream signaling pathway of smooth muscle cells. (**A**) Measurement of AChE activity. After homogenization of lung tissue, AChE activity was measured using an Acetylcholinesterase Assay Kit that could detect as little as 0.01 mU AChE in a 100 µL assay volume (0.1 mU/mL). (**B**) Detection of MLC phosphorylation. Expression of MLC and p-MLC measured using western blot analyses with an HRP-labeled secondary anti-rabbit IgG antibody, and (**C**) the relative levels of each protein was calculated relative to the intensity of actin bands. The data shown represent the means ± SD (*n* = 8). * indicates *p* < 0.05 compared to the No-treated group. # indicates *p* < 0.05 compared to the OVA + Vehicle-treated group.

To investigate the mechanism of the effects of BAW on cholinergic regulation of airway inflammation and remodeling, we measured changes in AChE activity and the AChR M3 downstream signaling pathway in airway tissue of an OVA-induced asthma model. After sensitization with OVA, the level of AChE activity was lower than that of the No-treated group. However, these levels increased remarkably in a dose-dependent manner in the OVA + BAWLo- and OVA + BAWHi-treated groups (Figure 7A). These results suggest that BAW can recover the secretion ability of ACh from neuronal and nonneuronal cells in the airway to prevent airway inflammation and remodeling.

In addition, recovery patterns observed in AChE activity were detected in the response of airway smooth muscle cells as target cells for ACh although, although they showed the opposite pattern. The phosphorylation level of MLC was higher in the OVA + Vehicle-treated group than the No-treated group. However, these levels were remarkably recovered in the OVA + BAW-treated group (Figure 7B,C). These results indicate that BAW-induced cholinergic recovery may be linked to regulation of the phosphorylation of MLC in smooth muscle cells.

**Figure 8:**
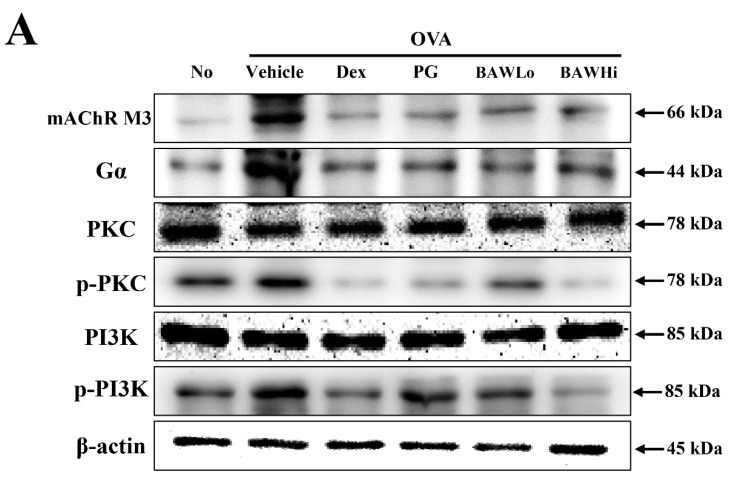

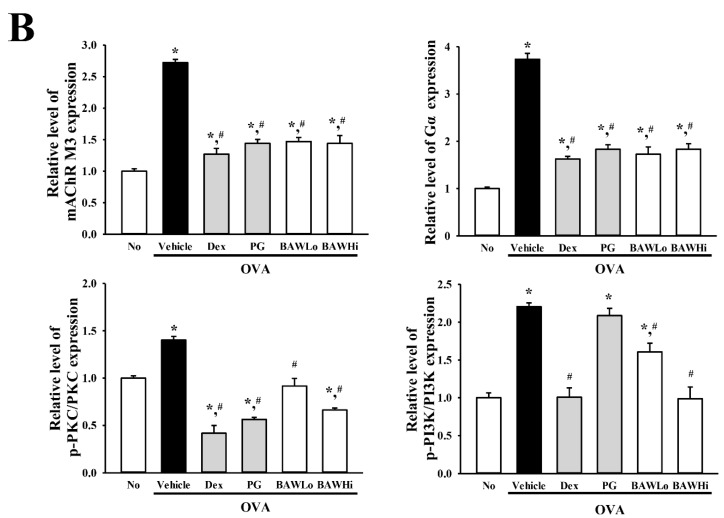
AChR M3 downstream signaling pathway in epithelial cells. (**A**) Expression of key components in the mAChR M3 downstream signaling pathway. Alterations in the expression of proteins related to the mAChR M3 signaling pathway were determined using western blot assays with an HRP-labeled anti-rabbit IgG antibody. (**B**) Band intensities were determined using an imaging densitometer, and the expression levels of the six proteins were evaluated relative to the intensity of actin bands. The data shown represent the means ± SD (*n* = 8). * indicates *p* < 0.05 compared to the No-treated group. # indicates *p* < 0.05 compared to the OVA + Vehicle-treated group.

Moreover, similar patterns were observed in the downstream signaling pathway of mAChR M3 in lung tissue, even though the range of alteration varied. After BAW treatment, the enhanced expression of mAChR M3, Gα, and phosphorylation of PKC and PI3K markedly recovered to those in the No-treated group (Figure 8A,B). However, alteration of the three signaling molecules as mAChR M3 downstream signaling factors did not fully reflect the activation of mAChR M3 because they were involved in various cellular responses in different cell types, including leukocytes. Nonetheless, these results show that the anti-asthmatic activity of BAW may be associated with the recovery of downstream signaling of mAChR M3 in lung tissue.

## 4. Discussion

Fermentation is a metabolic process in which a microorganism converts solid or liquid substances, such as starch or a sugar, into various products including an alcohol or an acid [28]. This technique is extensively applied to produce biomass, extracellular metabolites, and intracellular components, as well as to transform one specific substance into another [29]. Researchers are now focusing on identifying the pharmacologically active ingredients in fermented natural products to alleviate chronic diseases. Although there is a great deal of scientific evidence regarding the therapeutic effects of fermented products, only a few fermented products have been investigated for their anti-asthma effect in disease models [30,31]. Therefore, this study utilized the OVA-induced asthma model to examine the possibility that the fermented products of *A. cochinchinensis* roots could be potential anti-asthmatic agents. The results of the present study provide strong evidence that administration to BAW notably improves airway inflammation and remodeling in the lungs of OVA-induced asthma model mice and could therefore be considered a potential therapeutic drug for patients with allergic asthma.

Two previous reports have presented the relationship between fermented natural products and stimulation of anti-asthmatic parameters, although the analysis parameters were very limited. The treatment of *Artemisia princeps* Pampanini fermented with *Bifidobacterium infantis* K-525 showed the reduction of the IgE and cytokines levels in the trachea and lungs of experimental asthmatic mice [30]. Another study demonstrated that effective microorganism fermentation extract (EM-X) attenuated the airway hyper-reactivity and inflammatory response through suppression of leukocyte recruitment, as well as decreased Th2 cytokine levels and IgE concentrations in mice challenged with OVA [31]. These results are in agreement with those of the present study, in which airway inflammation was significantly alleviated after BAW treatment (250 and 500 mg/kg) for 6 days, although the natural product type and bacterial strain differed. However, the scope of analysis confirming the effectiveness of fermented products was more extensive in our study than in previous studies. This is because we investigated the effects related to lung histology in the current study, including the suppression of airway inflammation (including inflammatory cell infiltration and respiratory epithelium hyperplasia), as well as the inhibition of airway remodeling, such as excessive mucus production, collagen deposition, and angiogenesis. Furthermore, anti-asthmatic effects were higher in BAW than unfermented products of *A. cochinchinensis* when the relative effectiveness of quantitative indicators was compared. The rate of decrease of total cells (31%), macrophages (18%), and IL-4 concentration (79.3%) were greater in BAW than unfermented products of *A. cochinchinensis*, while the number of eosinophils, IgE level and thickness of respiratory epithelium remained constant in the same group. Taken together, these comparisons show that fermentation can be considered a technique to enhance the anti-asthmatic effects of *A. cochinchinensis*.

We further examined alterations in the influx of leukocytes, including total cells, eosinophils, and macrophages, where eosinophils are the major regulators of airway remodeling [32]. Eosinophil-mediated damage is a major mechanism underlying the pathogenesis of asthma, including airway inflammation and remodeling [33]. In the present study, there was a remarkable decrease in the total number of leukocytes (including lymphocytes, macrophages, and eosinophils) in the BALF of BAW-treated animals relative to the vehicle-treated group. Our finding indicates that BAW inhibits the influx of leukocytes during airway inflammation, which is consistent with the results of a previous study showing therapeutic effects of several fermented products including EM-X of unpolished rice, papaya, and seaweed in an OVA-induced asthma model in mice [31,34].

Alterations in the IgE and IL-4 levels are considered key markers of anti-asthmatic effects since the inflammatory response associated with asthma is characterized by infiltration of Th2 cells and leukocytes [35], and is characterized by elevated IgE in serum [1]. Among various Th2 cytokines, IL-4 has multiple immunological effects, including promotion of Th2 lineage differentiation, regulation of Ig class switching, and stimulation of mast cell proliferation [36]. Therefore, IgE and IL-4 levels are reportedly higher in OVA-induced asthma model mice than in normal controls [11]. These levels decreased significantly after treatment with fermented *Artemisia princeps* and EM-X, although there were some differences in the decrease ratio [30,31]. In this study, these two parameters dramatically decreased in the BALF and serum of OVA-induced asthma mice treated with BAW, which is consistent with the results of previous studies that showed the anti-asthmatic effects of fermented *Artemisia princeps* and EM-X. However, more studies are required to understand which key components determine the suppression of IgE and IL-4 production in each fermented product.

In this study, the increase in BAW dosage was correlated with suppression of airway remodeling in OVA-induced BALB/c mice. Several key factors are characteristic of airway remodeling, including thickening collagen layer, smooth muscle hyperplasia and enhancing VEGF expression. In the OVA-induced asthma model, a marked enhancement in the thickness of the collagen layer was observed and the α-SMA stained region within the airway [37,38]. Moreover, epithelial cell-derived VEGF is known to promote airway remodeling [39]. The hyperplasia of goblet cells and thickness of the basement membrane were significantly reduced when VEGF expression decreased [40]. In this study, there was a significant recovery of goblet cell hyperplasia, thickness of the collagen layer, and the VEGF signaling pathway in BAW-treated groups. These findings are similar to those of previous in vivo studies in which numerous natural products, including *Vitex rotundifolia* L. [41], *Astragali radix* Anti-Asthmatic Decoction [42], Bangpungtongseong-san [43], and Suhuang antitussive capsule [44], were reported to induce the stimulation of airway remodeling in OVA-treated BALB/c mice. However, the preventive effects of fermented natural products against airway remodeling have never been investigated in an OVA-induced asthma model. The results presented herein provide the first evidence that airway remodeling prevention of BAW may be linked to the regulation of goblet cell hyperplasia, collagen deposition, and angiogenesis.

Meanwhile, cholinergic regulation is considered an important factor in airway inflammation and remodeling during inflammatory lung diseases including asthma and COPD [45,46]. During this regulation, secretion of ACh from neuronal and nonneuronal cells exerts its functions through either mAChRs or nicotinic receptors [47]. mAChR have been shown to play a proinflammatory role in most structural cells of airway walls such as smooth muscle cells, fibroblasts, epithelial cells, and immune cells [45]. The expression level of mAChR M2 was reduced in airway neurons of asthma, while M1 and M3 levels were increased in airway structural cells of COPD patients [48,49,50]. Moreover, mAChR M3 mediated the effects of ACh in allergen-induced airway inflammation and remodeling of mice and guinea pigs [51,52]. The physiological and pathophysiological action of ACh, including bronchoconstriction, muscle thickening, and cytokine production, was observed in airway smooth muscle cells [53]. In this study, we investigated the action mechanism of BAW during the cholinergic regulation of airway inflammation and remodeling in an OVA-induced asthma model. A significant recovery of AChE activity and the mAChR M3 downstream signaling pathway was observed in epithelial cells and smooth muscle cells after BAW treatment. These results are the first to suggest that anti-inflammation and anti-remodeling activity of BAW may be associated with muscarinic cholinergic regulation in structural and inflammatory cells.

Furthermore, in this study, the unfermented product of *P. grandiflorum* was selected as a natural product with anti-asthmatic activity to compare the efficacy of BAW because only unfermented forms are commonly used to treat asthma in Korea [54,55]. Several extracts of unfermented roots of this plant effectively ameliorated bone marrow-derived mast cell-mediated allergy and OVA-induced asthma [56,57]. The aqueous extracts of *P. grandiflorum* inhibit the development of AD-like skin lesions in NC/Nga mice by inhibiting the Th2 cell response and stimulating the Th1 cell responses [58]. The greatest anti-asthmatic effects of BAW are observed at 500 mg/kg compared with 250 mg/kg for PG. However, the rate of decrease of OVA-specific IgE concentrations was higher than 70.7% in the OVA + BAWHi-treated group than the OVA + PG-treated group, but similar in the OVA + BAWLo-treated group (Figure 3C). Recovery of the expression of VEGF and the phosphorylation of MLC and PI3K were significantly greater in the BAW-treated (250 or 500 mg/kg) group than in the PG-treated (250 mg/kg) group. Therefore, our study shows the possibility that BAW can replace PG extracts for asthma treatment, although further analysis is needed to determine the therapeutic dosage and durability.

Taken together, the results of our study suggest that BAW attenuates airway inflammation and remodeling through cholinergic regulation in an OVA-induced asthma model in mice. The anti-asthmatic activities of BAW include inhibition of leukocyte influx, OVA-specific IgE production, Th2 cell activation, inflammatory cell infiltration, respiratory epithelial hyperplasia, goblet cell hyperplasia, angiogenesis, and collagen deposition. Cholinergic regulation in airways is associated with recovery of AChE activity and mAChR M3 downstream signaling. To the best of our knowledge, this study is the first to report that fermented products of *A. cochinchinensis* have anti-asthmatic activities and its functions are associated with cholinergic regulation using an OVA-induced asthma model. However, further studies are required to advance our understanding of the impending effects of BAW, as well as the molecular mechanisms responsible for these effects.
